# Prognostic factors of time to first abortion after sexual debut among fragile state Congolese women: a survival analysis

**DOI:** 10.1186/s12889-021-10599-x

**Published:** 2021-03-17

**Authors:** Michael Ekholuenetale, Charity Ehimwenma Ekholuenetale, Amadou Barrow

**Affiliations:** 1 Department of Epidemiology and Medical Statistics, Faculty of Public Health, College of Medicine, University of Ibadan, Ibadan, Nigeria; 2 Department of Economics, Benin Study Center, National Open University of Nigeria, Benin City, Nigeria; 3 Department of Public and Environmental Health, School of Medicine and Allied Health Sciences, University of The Gambia, Kanifing, The Gambia

**Keywords:** Pregnancy, Unwanted, Unintended, Termination, Unsafe abortion, Women’s health

## Abstract

**Background:**

Despite the common restrictive abortion laws, abortion remains widespread in sub-Saharan Africa (SSA) countries. Women still utilize abortion services and put their lives and health at risk because abortion can only be procured illegally in private facilities such as mid-level or small patent medicine store that may be manned by unskilled providers or through a non-medicated approach. The objective of this study was to investigate the prevalence of abortion, the reasons women had abortions, median years to first abortion after sexual debut and examine the factors of time to first abortion among women of reproductive age in the Republic of Congo.

**Methods:**

We used data from the most recent Republic of Congo Demographic and Health Survey (DHS). A total sample of 3622 women aged 15–49 years was analyzed. We estimated the overall prevalence of abortion and median years to first abortion. Furthermore, we examined the factors of time to first abortion after sexual debut using multivariable Cox regression and reported the estimates using adjusted Hazard Ratio (aHR) and 95% confidence intervals (CI). Statistical significance was determined at *p* < 0.05.

**Results:**

60% of pregnancies which are terminated are as a result of induced abortion and median years of time to first abortion after sexual debut was 9.0. The prominent reasons for abortion were due to too short birth interval (23.8%), lack of money (21.0%) and that husband/partner did not need a child at that time (14.0%). Women’s age and region were notable factors in timing to first abortion. Furthermore, women from poorer, middle, richer and richest households had 34, 67, 86 and 94% higher risk of abortion respectively, when compared with women from poorest households (all *p* < 0.05). Women currently in union/living with a man and formerly in union had 41 and 29% reduction in the risk of abortion respectively, when compared with those never in union (all *p* < 0.05). In addition, women with primary and secondary+ education had 42 and 76% higher risk of abortion respectively, when compared with women with no formal education (all *p* < 0.05).

**Conclusion:**

There was high prevalence of abortion with short years at first abortion. Abortion was associated with women’s characteristics. There is need for unwanted pregnancy prevention intervention and the improvement in pregnancy care to reduce adverse pregnancy outcomes among women.

## Background

The global maternal mortality ratio (MMR) reduced to 216 in 2015 from 385 deaths per 100,000 livebirths in 1990 [1]. The regional MMRs for 2015 ranged from 12 to 546 deaths per 100,000 livebirths for high-income regions and SSA respectively meaning that a high pregnancy-related death occurred in SSA [1]. Abortion is a known cause of maternal death [2]. It may be either safe or results in complications and death [3]. Abortion becomes unsafe when it is illegal and people cannot access safe services. Unsafe abortion accounts for over 60,000 maternal deaths annually [4], and causes disabilities in about 5 million women due to complications [5]. About 8% of maternal deaths was reported to be due to abortion and among those deaths, complications from unsafe abortion are one of the most common and easily preventable and curable causes [2, 6]. However, when performed by a trained provider in a legal setting, abortion is very safe. Reducing maternal mortality is a global health priority in the Sustainable Development Goals (SDGs) framework [7] and the global strategy for women and children’s health [8]. The objectives of global strategy include to end preventable deaths, ensure health and well-being and expand enabling environments.

Unintended pregnancies remain a major issue to be addressed in any abortion intervention. The higher unmet need for family planning remains, the more prevalent abortion becomes [9, 10]. Contraceptive use will in no small measure help vulnerable women to prevent the adverse effects of unsafe abortion, specifically in the instances of sexual violence, incest, child marriage and where having a pregnancy would endanger the life of a woman [11]. Unintended pregnancy has a substantial clinical, public and social health concerns, as it commonly leads to induced abortion and complications due to poor abortion services in many poor-resource countries [12]. The World Health Organization (WHO) reported that of the estimated 210 million pregnancies that occur annually, about 38% are unplanned and approximately 22% are terminated [13]. Over 200 million women in poor-resource settings would like to delay their next pregnancy or stop child birth altogether, unfortunately the majority of them still rely on less effective contraceptive methods [10].

Abortion is prohibited in many SSA countries in all circumstances including Gabon, Guinea-Bissau, Madagascar, Republic of Congo and Senegal [14, 15]. However, in some countries laws prohibit unlawful abortion but do not clearly specify what makes abortion lawful; The Gambia, Sierra Leone amongst others [15]. Albeit, the legal prohibition of induced abortion has not deterred women from terminating unwanted pregnancies [16]. The risk of criminal sanctions, has obliged them to do so without adequate medical care resulting in complications or death in some instances. Since the symptoms of spontaneous pregnancies termination renders a woman with early pregnancy distress suspicious of indulging in incomplete induced abortion, thereby susceptible to interrogations and mistreatment by medical personnel, women with spontaneous abortion would avoid seeking certain medical abortion services. Due to the restrictive abortion laws or cost for service, adolescent girls would commonly use plants with abortive properties as a strategy to abort a pregnancy secretly and at no cost, while even older women also rely on such methods to terminate unwanted pregnancies [17, 18].

The Republic of Congo has progressively improved in human development index (HDI). Congo’s HDI value for 2018 was 0.608 which put the country in the medium human development category - positioning it at 138 out of 189 countries and territories. Between 1990 and 2018, Congo’s HDI value increased from 0.531 to 0.608, an increase of 14.5% [19]. The 2018 female HDI value for Congo was 0.591 in contrast with 0.635 for males, resulting in a Gender Development Index (GDI) value of 0.931, placing it into Group 3 [19]. Poor gender power relations is attributable to inequities across most African countries including the Republic of Congo, resulting to women’s inability to make the decision of having a safe abortion [20, 21]. The Republic of Congo is on the WHO list of high burden countries for various health problems [22]. The issue of abortion is very critical because in the Republic of Congo, approximately 50% of maternal deaths occurs mainly in the intrapartum period [23]. This can be attributed as to why women would opt for abortion when they consider the magnitude of the risk of death during that period. This explains the high prevalence of pregnancy termination in the Republic of Congo (38.8%) [9].

In other SSA countries, the prevalence of pregnancy termination ranged from 7.5% in Benin to 39.5% in Gabon with an average of 16.5% [9]. In studies conducted among young women in African most populous country, up to half or more have terminated pregnancy, still the majority of them are against the legalization of abortion and rarely use contraceptive methods [24, 25]. Women are often unable to seek proper medical care in the advent of unintended pregnancies due to the fact that their culture and religion also prohibit abortion, yet widely practiced because of the stigmatization associated with an unwanted pregnancy in the community [26]. Although women make efforts to prevent unintended pregnancy, contraceptive failure contributes to a substantial proportion of unintended pregnancy [27]. Except for the modern contraceptive methods, many other available methods have low efficacy [27]. In light of the above, we aimed to investigate the prevalence of abortion, the reasons women had abortions, median years to first abortion after sexual debut and examine the factors of time to first abortion among women of reproductive age in the Republic of Congo.

## Methods

### Data sources

A cross-sectional data extracted from the Republic of Congo DHS 2012 was analyzed. A nationally representative sample of 3622 women who have had sex and aged 15–49 years were included in this study. On the other hand, the exclusion criterion was women with history of sexual abstinence. This was to ensure that only women exposed to pregnancy occurrence were analyzed. DHS data was collected through a stratified multistage cluster sampling technique. The procedure for stratification approach divides the population into groups by geographical region and commonly crossed by place of residence – urban vs. rural. A multi-level stratification approach is used to divide the population into first-level strata and to subdivide the first-level strata into second-level strata, and so on. DHS data is available in the public domain and accessed at; http://dhsprogram.com/data/available-datasets.cfm.

DHS has been conducted in over 85 countries and repeated every years since 1984. A major advantage is that the sampling design and data collection approach are similar across countries which makes the results of different settings comparable. Though from the onset, DHS was designed to expand on fertility, demographic and family planning data collected in the World Fertility Surveys and Contraceptive Prevalence Surveys, nonetheless, it has become the prominent source of population surveillance for the monitoring of population health indices particularly in resource-constrained settings. DHS elicits information from respondents in a wide range of health-related areas including vaccination, child and maternal mortality, fertility, intimate partner violence, female genital mutilation, nutrition, lifestyle, infectious and non-infectious diseases, family planning, water and sanitation amongst others. DHS has great merits in collecting high-quality data through proper interviewer training, national coverage, standardized data collection instrument and proper operational definition of concepts to enhance understanding among policy and decision makers. DHS data is useful in formulating epidemiological research to estimate prevalence, trends and inequalities. The details of DHS has been reported previously [28].

### Operational definition of variables

#### Outcome variable

The main outcome variable in this study was “abortion” also known as induced pregnancy termination. It was derived from the question; “Number of abortions” and responses were coded as “no” if a woman reported “0” indicating no history of abortion, and coded as “yes” if a woman reported “1”, “2”, “3”, “4” and so forth indicating history of abortion. In addition, the time to first abortion after sexual debut was also utilized. It was derived from the question; “Age at first abortion”. The difference in years between age at first abortion and “Age at first sex” was used as the time to first abortion. The main reason for abortion was derived from the question; “Main reason for putting an end to this pregnancy?” in the DHS individual woman dataset.

#### Explanatory variables

The factors examined in this study are based on previous studies related to abortion and presented in Table 1 below [9, 11, 30–32].

**Table 1:** Categories and operational definition of independent variables

	Variables	Definitions/categories
1	Age (in years)	Age of the respondent (15–19, 20–24, 25–29, 30–34, 35–39, 40–44, 45–49)
2	Geographical region	This is the region of residence for a respondent (Kouilou, Niari, Lekoumou, Bouenza, Pool, Plateaux, Cuvette, Cuvette-Ouest, Sangha, Likouala, Brazzaville, Pointe-Noire)
3	Residential status	Area of residence (urban, rural)
4	Wealth quintiles^a^	Economic/wealth status of the household (poorest, poorer, middle, richer, richest)
5	Health insurance coverage	Insured, uninsured
6	Marital status	Never in union, currently in union/living with a man, formerly in union/lived with a man
7	Exposure to media	This was generated from whether a respondent used any of newspaper/magazine, radio or television (yes, no)
8	Education	Respondent’s formal level of education/schooling (no formal education, primary, secondary/higher (secondary+)
9.	Religion	Protestant/Catholic, Islam, Spiritual
10	Ever used anything or tried to delay or avoid getting pregnant	The history of contraceptive use was measured dichotomously (yes, no)
11	Parity	Total number of children ever born (nil, 1–3, 4+)
12	Age (in years) at first birth	Age of a respondent when she had her first child (no birth, < 20, 20–24, 25+)
13	Age (in years) at first union/marriage	Age of a respondent when she entered a union/marriage (not married, < 20, 20–24, 25+)
14	Age at first sexual intercourse	Age of a respondent at sexual debut (< 18, 18+, at first union)
15	Total lifetime number of sex partners	Total number of men that a women had sexual relationship with (1–2, 3–4, 5+)

^a^For the calculation of household wealth status, household assets such as ownership of television, radio, bicycle possessed by the household and housing quality such as type of floor, wall and roof were taken into consideration. Each item is assigned a factor score generated through principal component analysis which are then summed and standardized for the households. These standardised scores places the households in a continuous scale based on relative wealth scores. The scores thus obtained from a continuous scale are subsequently categorised into quintiles to rank the household as poorest/poorer/middle/richer/richest to richest [29]

### Ethical consideration

We used publicly available data in this study. Since the data was not collected by the authors of this manuscript, we sought permission from MEASURE DHS/ICF International and access to the data was provided after our intent for the request was assessed and approved. MEASURE DHS Program is consistent with the standards for ensuring the protection of respondents’ privacy. ICF International ensures that the survey complies with the U.S. Department of Health and Human Services regulations for the respect of human subjects. No further approval was required for this study. More details about data and ethical standards are available at http://goo.gl/ny8T6X.

### Analytical approach

The survey (‘svy’) module was used to adjust for stratification, clustering and sampling weights to compute the estimates of abortion. To check multicollinearity, variance-inflation factor was employed and a value below 10 was considered acceptable [33, 34]. Consequently, no variable was excluded from the model as they were not found to be interdependent. We use percentage, Kaplan-Meier and Cox regression models to account for censoring in the estimation of exposure time to abortion [35, 36]. Statistical significance was determined at *p* < 0.05. Stata Version 14 (StataCorp., College Station, TX, USA) was used for data analysis.

## Results

### Summary statistics across women’s characteristics

Results from Table 2 showed an abortion prevalence of 60.0% and 9.0 years of median time to first abortion after sexual debut. Based on the results, women aged 15–19 years (63.3%), 20–24 years (67.8%) and 25–29 years (68.6%) had the highest history of abortion respectively. Women from Niari (79.0%) and Pointe-Neoire (76.3%) geographical region had the highest prevalence of abortion. Furthermore, the urban women (75.1%), women from the richest household (78.3%), insured (68.3%), never in union (77.5%), exposed to the media (66.7%), secondary/higher education (67.0%), had history of contraceptive use (63.1%), having 1–3 children (65.3%), below 20 years at first birth (62.6%), below 18 years at sexual debut (62.2%) and having 5+ total lifetime number of sex partners (67.8%) had the leading prevalence of abortion respectively. See Table 2 for the details.

**Table 2:** Distribution of abortion by women’s characteristics (*n* = 3622)

Variable	Distribution of respondents (%)	Median age at sexual debut	Median age at first abortion	Median years to first abortion	Percentage of pregnancies which are terminated as a result of induced abortion (%)
**Age (years)**					
15–19	210 (5.8)	15.0	16.0	1.0	63.3
20–24	528 (14.6)	15.0	19.0	3.0	67.8
25–29	749 (20.7)	15.0	21.0	5.0	68.6
30–34	641 (17.7)	15.0	24.0	8.0	62.4
35–39	630 (17.4)	15.0	26.0	10.0	56.8
40–44	461 (12.7)	15.0	23.0	7.0	46.9
45–49	403 (11.1)	15.0	23.0	7.5	47.4
**Region**					
Kouilou	422 (11.7)	15.0	22.0	7.0	71.8
Niari	238 (6.6)	15.0	21.0	5.0	79.0
Lekoumou	257 (7.1)	15.0	23.0	8.0	53.7
Bouenza	319 (8.8)	15.0	22.0	6.0	64.9
Pool	232 (6.4)	15.0	22.0	6.0	30.6
Plateaux	224 (6.2)	15.0	22.0	8.0	42.4
Cuvette	305 (8.4)	15.0	20.0	5.0	57.7
Cuvette-Ouest	242 (6.7)	15.0	21.0	5.0	39.7
Sangha	260 (7.2)	15.0	20.0	5.0	62.7
Likouala	256 (7.1)	15.0	21.0	6.0	35.2
Brazzaville	365 (10.1)	16.0	20.0	4.0	71.2
Pointe-Noire	502 (13.9)	16.0	20.0	5.0	76.3
**Residential status**					
Urban	1268 (35.0)	16.0	20.0	4.0	75.1
Rural	2354 (65.0)	15.0	22.0	6.0	51.7
**Wealth quintiles**					
Poorest	1236 (34.1)	15.0	22.0	8.0	41.8
Poorer	965 (26.6)	15.0	22.0	6.0	60.6
Middle	495 (13.7)	15.0	20.0	5.0	71.9
Richer	498 (13.8)	15.0	20.0	5.0	75.7
Richest	428 (11.8)	16.0	20.0	4.0	78.3
**Health insurance coverage**					
Insured	82 (2.3)	15.0	21.5	5.0	68.3
Uninsured	3537 (97.7)	15.0	21.0	5.0	59.7
**Marital status**					
Never in union	316 (8.7)	16.0	18.0	2.0	77.5
Currently in union/living with a man	2625 (72.5)	15.0	21.0	6.0	55.8
Formerly in union/lived with a man	681 (18.8)	15.0	22.0	6.0	67.6
**Exposure to media**					
Yes	2355 (65.0)	15.0	21.0	5.0	66.7
No	1267 (35.0)	15.0	21.0	6.0	47.4
**Education**					
No formal education	246 (6.8)	15.0	24.0	9.0	32.9
Primary	1195 (33.0)	15.0	22.0	7.0	52.6
Secondary+	2181 (60.2)	15.0	21.0	5.0	67.0
**Religion**					
Protestant/Catholic	1787 (49.4)	15.0	21.0	5.0	61.3
Islam	26 (0.7)	16.0	21.0	5.0	30.8
Spiritual	1806 (49.9)	15.0	21.0	6.0	59.0
**Ever used anything or tried to delay or avoid getting pregnant**					
Yes	2796 (77.2)	15.0	21.0	5.0	63.1
No	826 (22.8)	15.0	21.0	5.0	49.3
**Parity**					
Nil	300 (8.3)	16.0	18.0	2.0	59.7
1–3	1676 (46.3)	15.0	20.0	5.0	65.3
4+	1646 (45.4)	15.0	24.0	9.0	54.5
**Age at first birth**					
No birth	300 (8.3)	16.0	18.0	2.0	59.7
< 20	2205 (60.9)	15.0	21.0	6.0	62.6
20–24	879 (24.3)	16.0	22.0	6.0	56.2
25+	238 (6.6)	16.0	23.0	5.5	48.7
**Age at first marriage**					
Not married	316 (8.7)	16.0	18.0	2.0	77.5
< 20	2075 (57.3)	15.0	21.0	6.0	58.1
20–24	857 (23.7)	16.0	22.0	6.0	60.4
25+	374 (10.3)	16.0	23.0	7.0	54.0
**Age at first sexual intercourse**					
< 18	3003 (83.0)	15.0	21.0	6.0	62.2
18+	442 (17.0)	18.0	23.0	4.0	54.5
**Total lifetime number of sex partners**					
1–2	829 (23.5)	15.0	21.0	5.0	42.9
3–4	1307 (37.1)	15.0	21.0	5.0	61.4
5+	1389 (39.4)	15.0	21.0	6.0	67.8
**Total estimate**	**3622**	**15.0**	**21.0**	**9.0**	**60.0%**

### Main reason for abortion

**Fig. 1:**
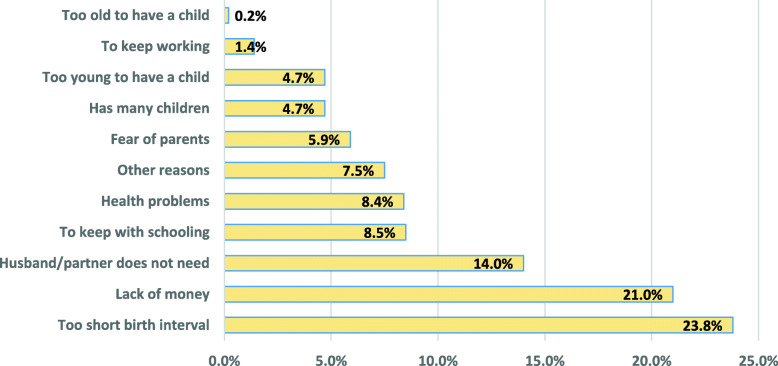
Main reason for putting an end to pregnancy

Figure 1 showed that the prominent reason for putting an end to pregnancy was due to too short birth interval (23.8%). Another major reason was due to lack of money (21.0%). Other reasons were that husband/partner did not need a child at that time it came (14.0%), to keep schooling (8.5%), health problems (8.4%), fear of parents (5.9%), many children (4.7%), too young to have a child (4.7%) respectively.

### Kaplan Meier plot for median time to first abortion after sexual debut

**Fig. 2:**
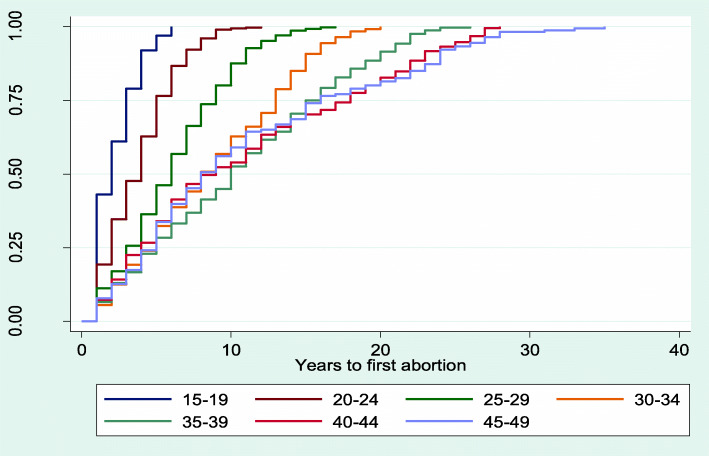
Kaplan-Meier failure estimates of time to first abortion by age

**Fig. 3:**
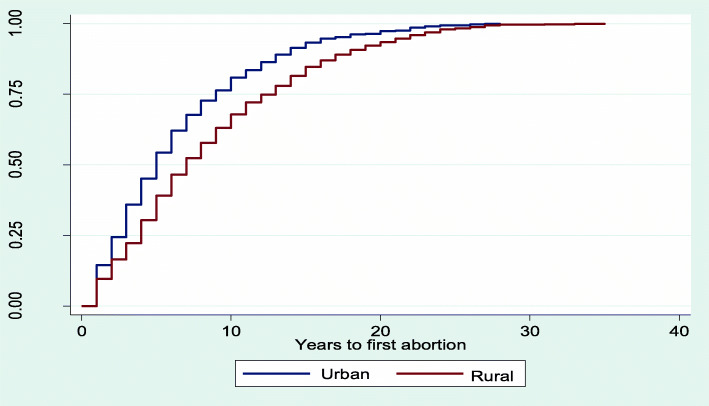
Kaplan-Meier failure estimates of time to first abortion by residence

**Fig. 4:**
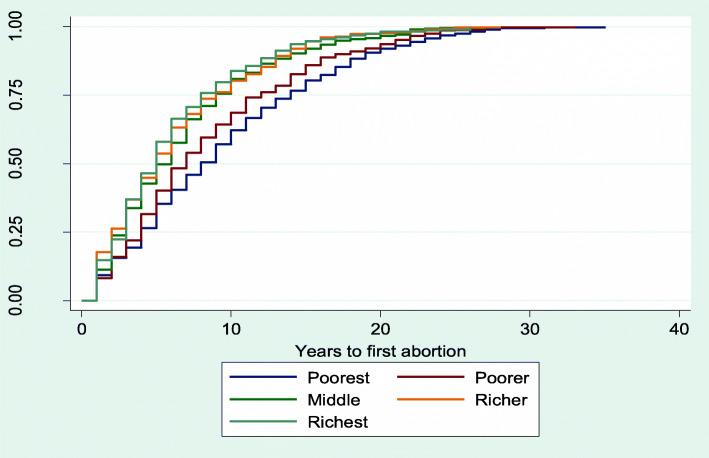
Kaplan-Meier failure estimates of time to first abortion by household wealth quintiles

**Fig. 5:**
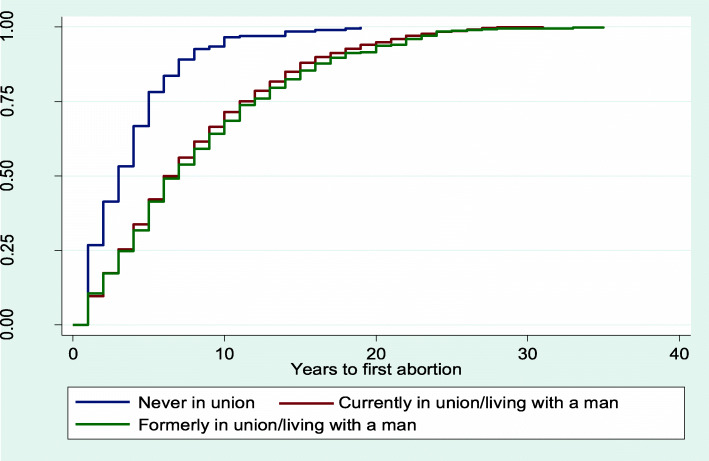
Kaplan-Meier failure estimates of time to first abortion by marital status

**Fig. 6:**
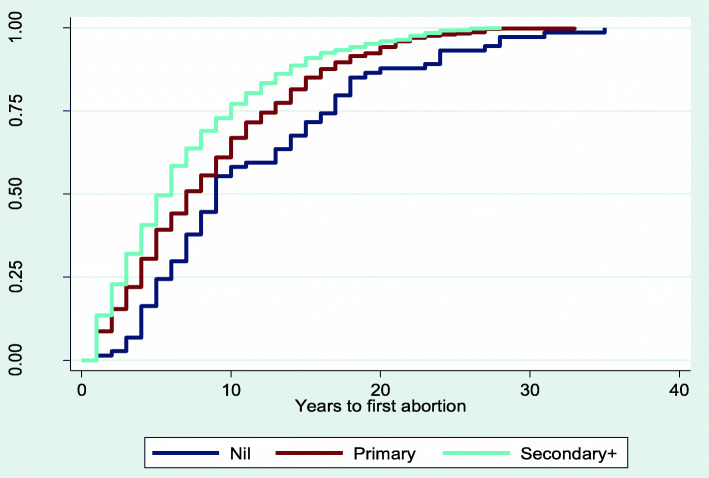
Kaplan-Meier failure estimates of time to first abortion by education

The results from Figs. 2, 3, 4, 5 and 6 showed that women aged 15–19 years and 20–24 years had the least median years to first abortion (Fig. 2). In addition, women of urban residence (Fig. 3), richest household (Fig. 4), never in union (Fig. 5), secondary+ education (Fig. 6) had the minimum median years to first abortion respectively.

**Fig. 7:**
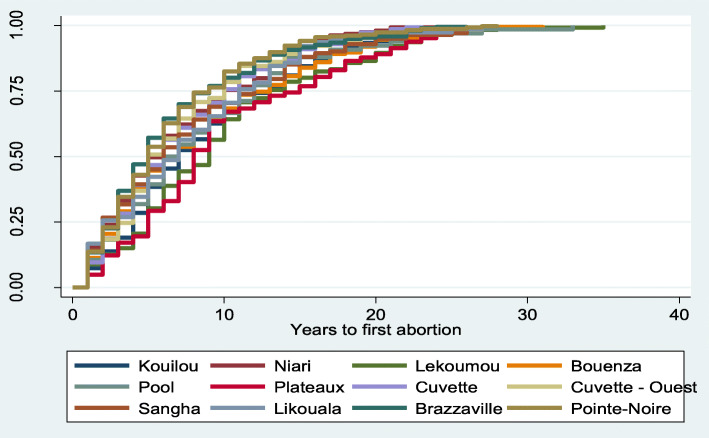
Kaplan-Meier failure estimates of time to first abortion by region

**Fig. 8:**
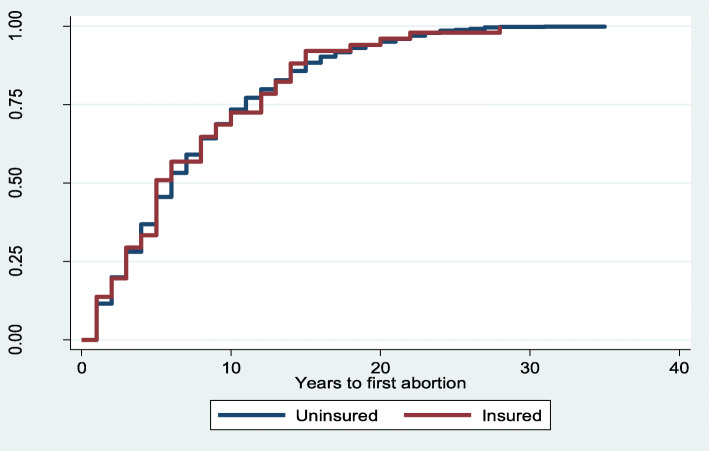
Kaplan-Meier failure estimates of time to first abortion by health insurance

**Fig. 9:**
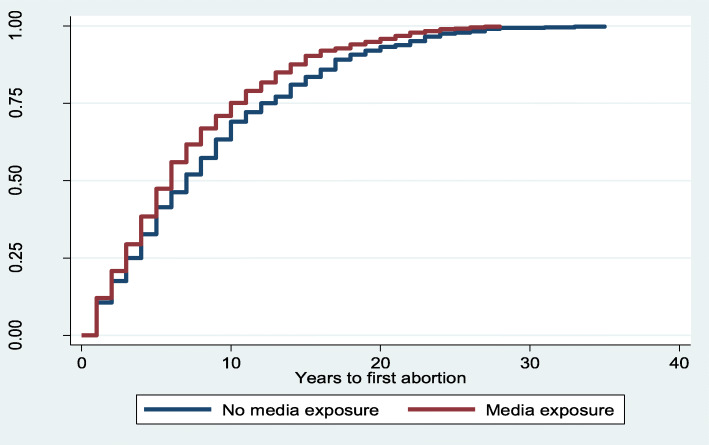
Kaplan-Meier failure estimates of time to first abortion by media use

**Fig. 10:**
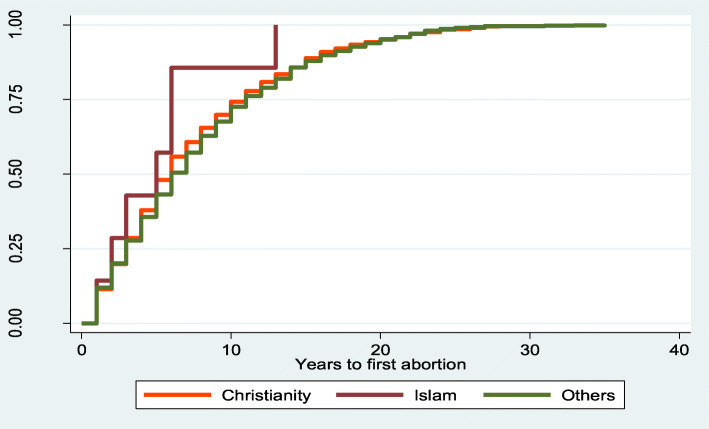
Kaplan-Meier failure estimates of time to first abortion by religion

**Fig. 11:**
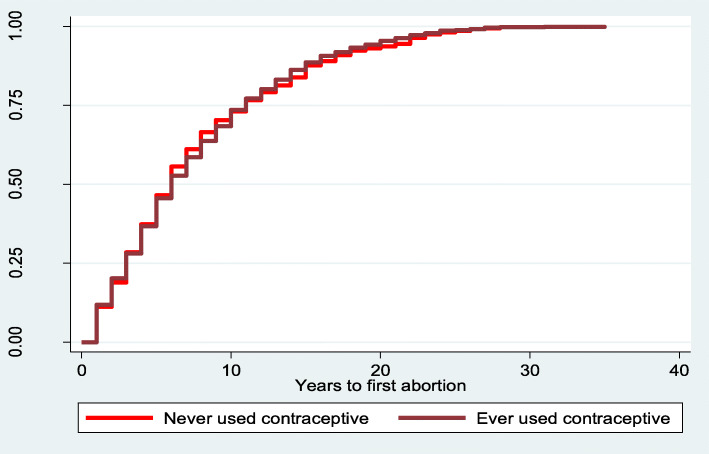
Kaplan-Meier failure estimates of time to first abortion by history of contraceptive use

**Fig. 12:**
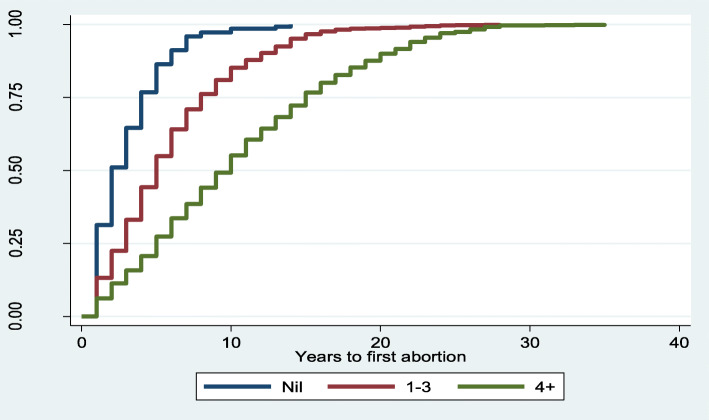
Kaplan-Meier failure estimates of time to first abortion by parity

**Fig. 13:**
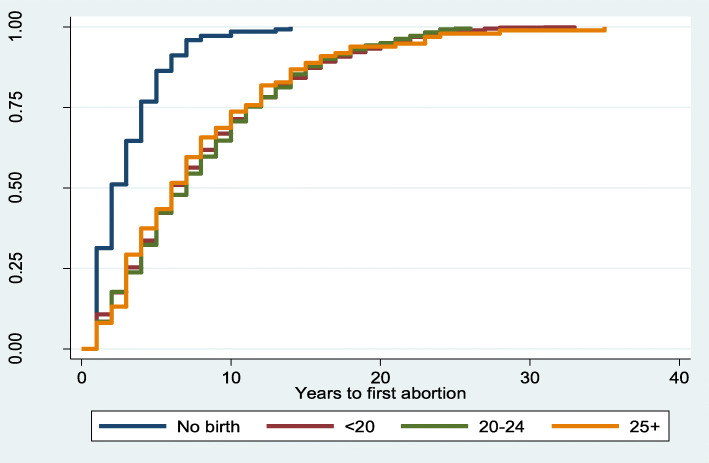
Kaplan-Meier failure estimates of time to first abortion by age at first birth

**Fig. 14:**
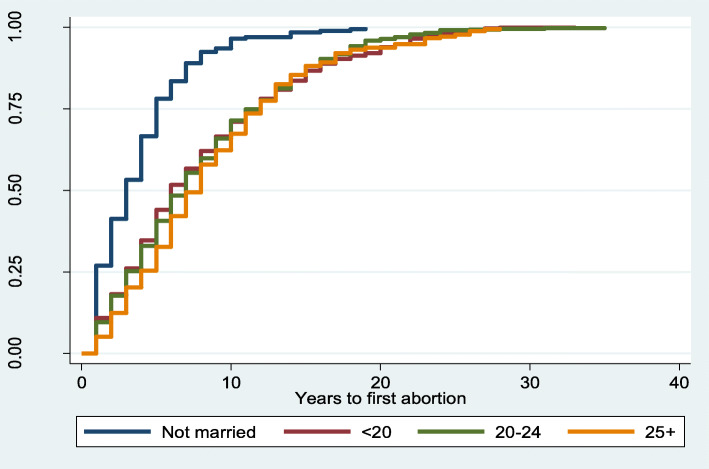
Kaplan-Meier failure estimates of time to first abortion by age at first marriage/union

**Fig. 15:**
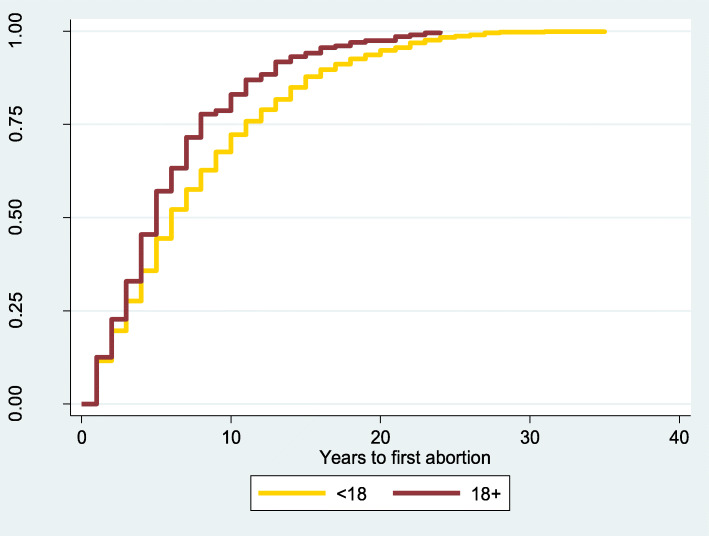
Kaplan-Meier failure estimates of time to first abortion by age at sexual debut

**Fig. 16:**
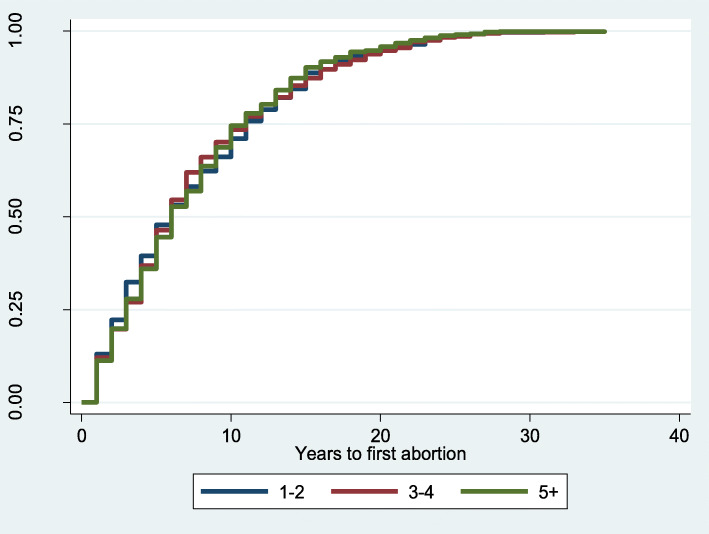
Kaplan-Meier failure estimates of time to first abortion by number of life time sexual partners

Figures 7, 8, 9, 10, 11, 12, 13, 14, 15 and 16 in Appendix showed the Kaplan-Meier failure estimates of time to first abortion by; region (Figure 7 in Appendix), health insurance coverage (Figure 8 in Appendix), media use (Figure 9 in Appendix), religion (Figure 10 in Appendix), history of contraceptive use (Figure 11 in Appendix), parity (Figure 12 in Appendix), age at first birth (Figure 13 in Appendix), age at first marriage/union (Figure 14 in Appendix), age at sexual debut (Figure 15 in Appendix) and the number of life time sexual partners (Figure 16 in Appendix) respectively.

### Prognostic factors of the time to first abortion

Table 3 showed the prognostic factors associated with time to first abortion among Congolese women of reproductive age. The risk of abortion after sexual debut was lower in the adjusted model among older women, when compared with young women aged 15–19 years. The geographical region was a significant determinant of abortion among Congolese women. Women from poorer, middle, richer and richest households had 34% (aHR = 1.34; 95%CI: 1.17, 1.53), 67% (aHR = 1.67; 95%CI: 1.40, 2.01), 86% (aHR = 1.86; 95%CI: 1.53–2.25) and 94% (aHR = 1.94; 95%CI: 1.57, 2.39) higher risk of abortion respectively, when compared with women from poorest households. In addition, women currently in union/living with a man and formerly in union had 41% (aHR = 0.59; 95%CI: 0.47, 0.75) and 29% (aHR = 0.71; 95%CI: 0.55, 0.91) lower risk of abortion respectively, when compared with women never in union. Furthermore, women with primary and secondary+ education had 42% (aHR = 1.42; 95%CI: 1.11, 1.81) and 76% (aHR = 1.76; 95%CI: 1.37, 2.25) higher risk of abortion respectively, when compared with women with no formal education. Women who have ever used anything or tried to delay or avoid getting pregnant had 37% (aHR = 1.37; 95%CI: 1.21, 1.54) higher risk of abortion, when compared with women who had never used any pregnancy prevention method. Those who had their first birth before age 20 years, had 32% (aHR = 1.32; 95%CI: 1.05, 1.65) higher risk of abortion, when compared with women with no child. Women having 3–4 and 5+ total lifetime number of sex partners had 28% (aHR = 1.28; 95%CI: 1.11, 1.47) and 49% (aHR = 1.49; 95%CI: 1.30, 1.72) higher risk of abortion respectively, when compared with women who had 1–2 total lifetime number of sex partners.

**Table 3:** Factors associated with the time to first abortion after sexual debut

Variable	Unadjusted Hazard Ratio	95% CI	Adjusted Hazard Ratio	95% CI
**Age (years)**				
15–19	Reference		Reference	
20–24	0.55	0.44–0.69*	0.53	0.42–0.68*
25–29	0.34	0.27–0.43*	0.34	0.26–0.43*
30–34	0.20	0.16–0.25*	0.21	0.16–0.27*
35–39	0.14	0.11–0.18*	0.15	0.12–0.20*
40–44	0.09	0.07–0.12*	0.10	0.08–0.14*
45–49	0.09	0.07–0.12*	0.10	0.08–0.14*
**Region**				
Kouilou	Reference		Reference	
Niari	1.37	1.13–1.66*	0.66	0.51–0.85*
Lekoumou	0.63	0.51–0.77*	0.50	0.41–0.63*
Bouenza	0.93	0.77–1.11	0.75	0.61–0.92*
Pool	0.32	0.25–0.42*	0.33	0.25–0.44*
Plateaux	0.51	0.40–0.65*	0.45	0.34–0.58*
Cuvette	0.81	0.66–0.98*	0.56	0.45–0.69*
Cuvette-Ouest	0.43	0.33–0.56*	0.34	0.26–0.44*
Sangha	0.87	0.72–1.07	0.46	0.36–0.58*
Likouala	0.45	0.35–0.58	0.35	0.27–0.45*
Brazzaville	1.15	0.97–1.37	0.55	0.42–0.73*
Pointe-Noire	1.38	1.18–1.61*	0.63	0.49–0.82*
**Residential status**				
Urban	Reference		Reference	
Rural	0.50	0.46–0.54*	0.99	0.81–1.23
**Wealth quintiles**				
Poorest	Reference		Reference	
Poorer	1.67	1.48–1.90*	1.34	1.17–1.53*
Middle	2.56	2.21–2.96*	1.67	1.40–2.01*
Richer	2.80	2.43–3.22*	1.86	1.53–2.25*
Richest	2.95	2.55–3.43*	1.94	1.57–2.39*
**Health insurance coverage**				
Insured	1.20	0.90–1.58		
Uninsured	Reference			
**Marital status**				
Never in union	Reference		Reference	
Currently in union/living with a man	0.33	0.28–0.38*	0.59	0.47–0.75*
Formerly in union/lived with a man	0.42	0.35–0.50*	0.71	0.55–0.91*
**Exposure to media**				
Yes	1.72	1.56–1.91*	1.12	0.99–1.26
No	Reference		Reference	
**Education**				
No formal education	Reference		Reference	
Primary	1.70	1.34–2.17*	1.42	1.11–1.81*
Secondary+	2.59	2.05–3.26*	1.76	1.37–2.25*
**Religion**				
Protestant/Catholic	Reference			
Islam	0.60	0.28–1.25		
Spiritual	0.97	0.89–1.06		
**Ever used anything or tried to delay or avoid getting pregnant**				
Yes	1.44	1.29–1.61*	1.37	1.21–1.54*
No	Reference		Reference	
**Parity**				
Nil	Reference		Reference	
1–3	0.71	0.60–0.85*	0.94	0.71–1.24
4+	0.38	0.31–0.45*	0.92	0.67–1.26
**Age at first birth**				
No birth	Reference		Reference	
< 20	0.56	0.47–0.66*	1.32	1.05–1.65*
20–24	0.47	0.39–0.57*	1.13	0.90–1.42
25+	0.35	0.27–0.45*	1.00	–
**Age at first marriage**				
Not married	Reference		Reference	
< 20	0.36	0.31–0.42*	0.94	0.79–1.13
20–24	0.36	0.30–0.42*	1.04	0.87–1.26
25+	0.28	0.23–0.34*	1.00	–
**Age at first sexual intercourse**				
< 18	Reference			
18+	0.90	0.70–1.04		
**Total lifetime number of sex partners**				
1–2	Reference		Reference	
3–4	1.43	1.24–1.63*	1.28	1.11–1.47*
5+	1.63	1.42–1.86*	1.49	1.30–1.72*

*Significant at *p* < 0.05

## Discussion

This is the foremost study in the Republic of Congo to explore the prevalence of abortion, median years to abortion after sexual debut and the prognostic factors of time to first abortion. The findings showed that about 60% of women had history of abortion. This is higher than 38.8% previously reported from the country [9] and pooled prevalence for Africa (3.9%) [37]. Similarly, higher than the prevalence of abortion in Nigeria (11%) [11], Mozambique (9%) and Ghana (25%) [30], Benin (7.5%), Gabon (39.5%) and Democratic Republic of Congo (18.5%) [9] respectively. Notably, there were differences between this study’s findings and a previous report in the Congo, being from the same data set such as the prevalence of abortion (23%) [38]. This difference could be due to the sub-sample of the original data set extracted for this study. While we expected the prevalence of abortion to be lower in this population given gender power imbalances that typically reduce women’s ability to make autonomous reproductive health decisions, we actually observed a very high prevalence of abortion. This might be because of secondary education as more women had at least secondary education. Educational attainment will aid a woman to identify pregnancy-related danger signs and what to do with it. In a previous study, education was identified as a key factor to explain not only an induced abortion decision but also having a repeated or a second trimester induced abortion [39]. Another reason could be that women do not have much reproductive decision-making autonomy and, therefore, might not be able to negotiate contraceptive use [40]. In turn, there are unintended pregnancies that women must terminate through abortion particularly due to very young age or when the partner is not able to take responsibility [41].

Pregnancy termination is one of the key issues that require urgent attention to achieve the third SDG of ensuring healthy lives and promoting wellbeing for all at all ages [7]. The reproductive health decision-making capacity of women plays a key role in their reproductive health outcomes, including pregnancy termination. Unsafe abortion is unavoidable in a society where laws, culture, religion amongst others prohibit the practice thereby leading to restrictions on access to safe and legal abortion [5].

There are numerous reasons why women undertake abortion. In line with the findings of this study, women gave reasons such as wrong timing, which encompassed a sense of not being ready for motherhood and the desire not to disrupt education, work, or life plans and because of health problems as also reported from a previous study [42]. Other reasons for seeking abortion-related to life circumstances, including cost, readiness, not wanting any more children, marital issues or relationship instability, and being too young in line with results from a previous study [43]. In addition, being too young to be a mother is an aspect of being unready for motherhood. This is consistent with the findings from previous studies [43, 44]. Prominently, husband/partner not needing a child contributed to reasons for abortion in our study. This could be either by his absence or because the relationship was troubled, unstable, too new, or not being in a relationship at all, the partner’s immaturity, his unreliability, his reluctance to be a father, the women’s reluctance to have him as her baby’s father, his unfaithfulness, drinking, physical abuse, and problems with paternity (such as the partner’s denial of paternity or pregnancy to someone other than the partner). Furthermore, women’s financial problems and very short preceding birth interval were part of the major reasons that contributed to women’s sense that they would be unable to care adequately for a child and that abortion was the most responsible action to take unfortunately. These findings are similar to report from a previous study which stated the most frequently cited reasons for having an abortion in most countries were socioeconomic concerns or limiting childbearing [45].

Overall, the median years between sexual debut and first abortion was 9.0. However, there were differences in years between sexual debut and first abortion across women’s characteristics. For example, the adolescent women (15–19 years) and those aged 20–24 years had the lowest median years to first abortion respectively, when compared with older women. This finding is consistent with the results of a previous study [46]. In that study, the difference between age at first intercourse and age at onset of first pregnancy was 1.3 years among adolescents compared to 4.8 years for older women. Also, the study confirmed that young women were more at risk of an unwanted pregnancy soon after menarche [46]. It is common that societal, social, family and career pressure will make the young women to opt for abortion from unwanted pregnancy. Furthermore, women of high educational attainment and household wealth quintiles had lower median years between sexual debut and first abortion. As reported by the women, some reasons for abortion were to keep with schooling or working. Consequently, education and wealth will enhance the decision to opt for abortion in the advent of unwanted pregnancy.

In this study, the geographical region was a significant factor of first abortion. This could be due to regional differences in strength of health district officers and programs. It is possible that various districts have more resources and health care infrastructure than others, or perhaps different regions have different sociocultural norms around gender equality and reproduction. Reports from previous studies showed regional differences in the uptake and utilization of health care services [47–51]. In addition, older women and those who were married had a reduction in the risk of first abortion. Furthermore, women from higher households wealth quintiles, educated, those who had ever used anything or tried to delay or avoid getting pregnant, had their first birth before age 20 years and having higher total lifetime number of sex partners had higher risk of abortion among Congolese women. Although, there was no previous study identified anywhere in the world that examined the prognostic factors of time to first abortion, however, these findings are comparable with the results from previous studies that examined the factors associated with history of abortion [9, 11, 30–32]. Women of higher socioeconomic status will usually weigh the options on whether to keep a pregnancy or not, as such could have higher risk of first abortion. The pursuit of career such as schooling or working, can make a woman take a decision to terminate a pregnancy. On the other hand, women in high socioeconomic status would have the knowledge and economic empowerment to afford abortion services. In addition, women with high total lifetime number of sex partners are more likely to be sexually active over time, and if effective contraceptive method is not utilized, more unwanted pregnancies can occur which could lead to higher risk of abortion.

Conversely, our finding on older women having reduction in the risk of first abortion, was inconsistent with the result from previous studies where older women had increased odds of pregnancy termination history [9, 11, 30]. Notably, our outcome variable of time to first sex after sexual debut is somewhat different from the outcome variable (history of abortion) of the previous studies used for comparison. Based on our findings, it is correct to say that the older women would have more awareness of the methods to delay getting pregnant after sexual debut. Moreover, women who were married/living with partner would have less risk of first abortion because unlike the single women, the married counterparts are able to keep pregnancy for the need of the family. Furthermore, early childbearing especially beginning from adolescence would mean that a woman would have large number of children long before the menopause stage. The implication is that the woman may now resort to the use of contraceptive methods or terminating further pregnancies especially in the advent of economic hardship or limited resources. Consequently, such women will have higher risk of abortion. The risk of abortion was higher among women who ever used anything or tried to delay or avoid getting pregnant. The issue is prominently hinged on the type of contraceptive methods. Moreover, it is possible that contraceptive use was introduced to the women during post-abortion care service or as a lesson learned. Sexually active women who do not use modern contraceptive methods are more likely to have unwanted pregnancy and consequently have higher risk of abortion. This is consistent with the findings of a previous study where women who had unintended pregnancy were more likely to terminate pregnancy [11]. It is not sufficient to use any contraceptive methods, the emphasis should be using effective contraceptive methods.

### Strengths and limitations

A major strength of this study was the use of nationally representative data and the findings are generalisable for the women of reproductive age. In addition, relevant variables were available in the datasets for the estimation of time to first abortion after sexual debut. Nonetheless, the study captured the life time experience of women on abortion, the occurrence of recall bias is very likely as highly multigravida women could have lost track of certain sexual and reproductive events, thereby leading to underestimation of abortion. Moreover, caution should be taken in using the high prevalence of abortion, as the number of women included in this study was only about one-thirds of the original sample size analyzed by DHS with less abortion prevalence.

## Conclusion

We identified a high prevalence of abortion with short years interval between age at sexual debut and time at first abortion. Abortion was associated with several women’s characteristics. These findings underline the importance of the further implementation of pregnancy prevention strategies and the improvement of healthcare interventions to reduce adverse birth outcomes. We recommend extensive and multidisciplinary research to explore the possibilities and reasons for abortion among Congolese women despite the prohibitive laws. Though the Republic of Congo government has not legalised the practice of abortion, evidence suggests that it continues to be prevalent and that the government needs to take steps to make abortion safer for women. Besides, the government may want to provide legal ground for abortion when the pregnancy endangers the life of a woman, in the instance of sexual violence including incest. Moreover, increased efforts are also needed to prevent unintended pregnancy in its entirety and to reduce levels of unsafe abortion and its negative health, social and economic effects.

## Data Availability

Data for this study were sourced from Demographic and Health surveys (DHS) and available here: http://dhsprogram.com/data/available-datasets.cfm.

## Abbreviations

CI: Confidence Intervals
DHS: Demographic and Health Survey
GDI: Gender Development Index
HR: Hazard Ratio
HDI: Human Development Index
ICF: Inner City Fund
MMR: Maternal mortality ratio
SDGs: Sustainable Development Goals
SSA: Sub-Saharan Africa
TX: Texas
US: United States
WHO: World Health Organization

## References

[1] Alkema L, Chou D, Hogan D, Zhang S, Moller A-B, Gemmill A, Fat DM, Boerma T, Temmerman M, Mathers C, Say L, United Nations Maternal Mortality Estimation Inter-Agency Group collaborators and technical advisory group (2016). Global, regional, and national levels and trends in maternal mortality between 1990 and 2015, with scenario-based projections to 2030: a systematic analysis by the UN Maternal Mortality Estimation Inter-Agency Group. Lancet.

[2] Say L, Chou D, Gemmill A, Tunçalp Ö, Moller A-B, Daniels J, Gülmezoglu AM, Temmerman M, Alkema L (2014). Global causes of maternal death: a WHO systematic analysis. Lancet Glob Health.

[3] Yogi A, Prakash KC, Neupane S (2018). Prevalence and factors associated with abortion and unsafe abortion in Nepal: a nationwide cross-sectional study. BMC Pregnancy Childbirth.

[4] Rasch V (2011). Unsafe abortion and postabortion care - an overview. Acta Obstet Gynecol Scand.

[5] Shah I, Åhman E (2009). Unsafe abortion: global and regional incidence, trends, consequences, and challenges. J Obstet Gynaecol Can.

[6] Kigbu JH, Daru PH, Ujah I a O (2009). Review of maternal deaths from unsafe abortion in Jos, Nigeria. Niger J Med J Natl Assoc Resid Dr Niger.

[7] Rosa W, editor. Transforming our world: the 2030 agenda for sustainable development. In: A new era in global health. New York: Springer Publishing Company; 2017. doi:10.1891/9780826190123.ap02.

[8] Kuruvilla S, Bustreo F, Kuo T, Mishra C, Taylor K, Fogstad H, Gupta GR, Gilmore K, Temmerman M, Thomas J, Rasanathan K, Chaiban T, Mohan A, Gruending A, Schweitzer J, Dini HS, Borrazzo J, Fassil H, Gronseth L, Khosla R, Cheeseman R, Gorna R, McDougall L, Toure K, Rogers K, Dodson K, Sharma A, Seoane M, Costello A (2016). The global strategy for women’s, children’s and adolescents’ health (2016–2030): a roadmap based on evidence and country experience. Bull World Health Organ.

[9] Seidu A-A, Ahinkorah BO, Ameyaw EK, Hubert A, Agbemavi W, Armah-Ansah EK, Budu E, Sambah F, Tackie V (2020). What has women’s reproductive health decision-making capacity and other factors got to do with pregnancy termination in sub-Saharan Africa? Evidence from 27 cross-sectional surveys. PLoS One.

[10] Peterson HB, Darmstadt GL, Bongaarts J (2013). Meeting the unmet need for family planning: now is the time. Lancet.

[11] Yaya S, Amouzou A, Uthman OA, Ekholuenetale M, Bishwajit G, Udenigwe O, Hudani A, Shah V (2018). Prevalence and determinants of terminated and unintended pregnancies among married women: analysis of pooled cross-sectional surveys in Nigeria. BMJ Glob Health.

[12] Ganatra B, Gerdts C, Rossier C, Johnson BR, Tunçalp Ö, Assifi A (2017). Global, regional, and subregional classification of abortions by safety, 2010–14: estimates from a Bayesian hierarchical model. Lancet.

[13] World Health Organization (2012). Safe abortion: technical and policy guidance for health systems.

[14] Bearak J, Popinchalk A, Ganatra B, Moller A-B, Tunçalp Ö, Beavin C, Kwok L, Alkema L (2020). Unintended pregnancy and abortion by income, region, and the legal status of abortion: estimates from a comprehensive model for 1990–2019. Lancet Glob Health.

[15] Lavelanet AF, Schlitt S, Johnson BR, Ganatra B (2018). Global abortion policies database: a descriptive analysis of the legal categories of lawful abortion. BMC Int Health Hum Rights.

[16] Worrell M. Abortion laws Gabon. Women on waves. http://www.womenonwaves.org/en/page/5189/abortion-laws-gabon. Accessed 4 Aug 2020.

[17] Pick De Weiss S, David HP (1990). Illegal abortion in Mexico: client perceptions. Am J Public Health.

[18] Mundigo AI, Indriso C, World Health Organization (1999). Abortion in the developing world.

[19] UNDP. Inequalities in human development in the 21st century briefing note for countries on the 2019 human development report. http://hdr.undp.org/sites/all/themes/hdr_theme/country-notes/COG.pdf. Accessed 27 Oct 2020.

[20] Patel CJ, Kooverjee T (2009). Abortion and contraception: attitudes of South African university students. Health Care Women Int.

[21] Nwogwugwu N, Yacob-Haliso O, Falola T (2019). Women’s empowerment and women’s health in Africa. The Palgrave handbook of African women’s studies.

[22] Linguissi LSG, Gwom LC, Nkenfou CN, Bates M, Petersen E, Zumla A, Ntoumi F (2017). Health systems in the Republic of Congo: challenges and opportunities for implementing tuberculosis and HIV collaborative service, research, and training activities. Int J Infect Dis.

[23] Merdad L, Ali MM (2018). Timing of maternal death: levels, trends, and ecological correlates using sibling data from 34 sub-Saharan African countries. PLoS One.

[24] Oriji VK, Jeremiah I, Kasso T (2009). Induced abortion amongst undergradute students of University of Port Harcourt. Niger J Med J Natl Assoc Resid Dr Niger.

[25] Moronkola OA, Amosu A, Okonkwo C (2005). Knowledge about conception, sexual behavior, and procurement of abortion among female undergraduate students in a Nigerian university. Int Q Community Health Educ.

[26] Omo-Aghoja LO, Omo-Aghoja VW, Okonofua FE, Aghedo O, Umueri C, Otayohwo R, Feyi-Waboso P, Esume CO (2009). Perceptions and attitudes of a rural community to abortion in the Niger-delta region of Nigeria. Niger J Clin Pract.

[27] Black KI, Gupta S, Rassi A, Kubba A (2010). Why do women experience untimed pregnancies? A review of contraceptive failure rates. Best Pract Res Clin Obstet Gynaecol.

[28] Corsi DJ, Neuman M, Finlay JE, Subramanian S (2012). Demographic and health surveys: a profile. Int J Epidemiol.

[29] Rutstein SO, Staveteig S (2014). Making the demographic and health surveys wealth index comparable. DHS methodological reports no. 9.

[30] Dickson KS, Adde KS, Ahinkorah BO (2018). Socio – economic determinants of abortion among women in Mozambique and Ghana: evidence from demographic and health survey. Arch Public Health.

[31] Tilahun F, Dadi AF, Shiferaw G (2017). Determinants of abortion among clients coming for abortion service at felegehiwot referral hospital, northwest Ethiopia: a case control study. Contracept Reprod Med.

[32] Ratovoson R, Kunkel A, Rakotovao JP, Pourette D, Mattern C, Andriamiadana J, Harimanana A, Piola P (2020). Frequency, risk factors, and complications of induced abortion in ten districts of Madagascar: results from a cross-sectional household survey. BMC Womens Health.

[33] García CB, García J, Martín MML, Salmerón R (2015). Collinearity: revisiting the variance inflation factor in ridge regression. J Appl Stat.

[34] Thompson CG, Kim RS, Aloe AM, Becker BJ (2017). Extracting the variance inflation factor and other multicollinearity diagnostics from typical regression results. Basic Appl Soc Psychol.

[35] Fagbamigbe AF, Akintayo AO, Oshodi OC, Makinde FT, Babalola M, Araoye ED, Enabor OC, Dairo MD (2020). Survival analysis and prognostic factors of time to first domestic violence after marriage among Nigeria, Kenya, and Mozambique women. Public Health.

[36] Ekholuenetale M, Wegbom AI, Tudeme G, Onikan A (2020). Household factors associated with infant and under-five mortality in sub-Saharan Africa countries. Int J Child Care Educ Policy.

[37] Khan KS, Wojdyla D, Say L, Gülmezoglu AM, Look PFV (2006). WHO analysis of causes of maternal death: a systematic review. Lancet.

[38] Centre Nationale de la Statistique et des Études Économiques (CNSEE) [Congo] et ICF International. 2013 Enquête Démographique et de Santé du Congo (EDSC-II) 2011-2012. Calverton: CNSEE et ICF International. https://dhsprogram.com/pubs/pdf/FR267/FR267.pdf. Accessed 4 Mar 2021.

[39] González-Rábago Y, Rodriguez-Alvarez E, Borrell LN, Martín U (2017). The role of birthplace and educational attainment on induced abortion inequalities. BMC Public Health.

[40] Yaya S, Uthman OA, Ekholuenetale M, Bishwajit G (2018). Women empowerment as an enabling factor of contraceptive use in sub-Saharan Africa: a multilevel analysis of cross-sectional surveys of 32 countries. Reprod Health.

[41] Cleeve A, Faxelid E, Nalwadda G, Klingberg-Allvin M. Abortion as agentive action: reproductive agency among young women seeking post-abortion care in Uganda. Cult Health Sex. 2017;19:1286–300. 10.1080/13691058.2017.1310297. 10.1080/13691058.2017.1310297 28398161

[42] Kirkman M, Rowe H, Hardiman A, Mallett S, Rosenthal D (2009). Reasons women give for abortion: a review of the literature. Arch Womens Ment Health.

[43] Santelli JS, Speizer IS, Avery A, Kendall C (2006). An exploration of the dimensions of pregnancy intentions among women choosing to terminate pregnancy or to initiate prenatal care in New Orleans, Louisiana. Am J Public Health.

[44] Bm H, Christensson K, Olsson P (2005). Meanings of being pregnant and having decided on abortion: young Swedish women’s experiences. Health Care Women Int.

[45] Chae S, Desai S, Crowell M, Sedgh G (2017). Reasons why women have induced abortions: a synthesis of findings from 14 countries. Contraception.

[46] Schor N (1993). Abortion and adolescence: relation between the menarche and sexual activity. Int J Adolesc Med Health.

[47] Camenzind PA (2012). Explaining regional variations in health care utilization between Swiss cantons using panel econometric models. BMC Health Serv Res.

[48] Johansson N, Jakobsson N, Svensson M (2018). Regional variation in health care utilization in Sweden – the importance of demand-side factors. BMC Health Serv Res.

[49] Laksono AD, Rukmini R, Wulandari RD (2020). Regional disparities in antenatal care utilization in Indonesia. PLoS One.

[50] Tanou M, Kamiya Y (2019). Assessing the impact of geographical access to health facilities on maternal healthcare utilization: evidence from the Burkina Faso demographic and health survey 2010. BMC Public Health.

[51] Okoli C, Hajizadeh M, Rahman MM, Khanam R (2020). Geographical and socioeconomic inequalities in the utilization of maternal healthcare services in Nigeria: 2003–2017. BMC Health Serv Res.

