# Correction: Prognostic factors of time to first abortion after sexual debut among fragile state Congolese women: a survival analysis

**DOI:** 10.1186/s12889-024-18697-2

**Published:** 2024-05-15

**Authors:** Michael Ekholuenetale, Charity Ehimwenma Ekholuenetale, Amadou Barrow

**Affiliations:** 1 Department of Epidemiology and Medical Statistics, Faculty of Public Health, College of Medicine, University of Ibadan, Ibadan, Nigeria; 2 Department of Economics, Study Center, National Open University of Nigeria, Benin, Nigeria; 3 Department of Public and Environmental Health, School of Medicine and Allied Health Sciences, University of The Gambia, Kanifing, The Gambia

**BMC Public Health (2021) 21:525** 10.1186/s12889-021-10599-x

In the original publication there was an error in interpreting the prevalence of abortion. The prevalence of abortion should read: “60% of pregnancies which are terminated are as a result of induced abortion”, instead of: “prevalence of abortion was 60.0%”. This correction also applies to: “Percentage history of abortion (%) in Table 2”, it rather refers to “percentage of pregnancies which are terminated as a result of induced abortion”. The original article has been updated.

